# LTP Promotes a Selective Long-Term Stabilization and Clustering of Dendritic Spines

**DOI:** 10.1371/journal.pbio.0060219

**Published:** 2008-09-09

**Authors:** Mathias De Roo, Paul Klauser, Dominique Muller

**Affiliations:** Department of Neuroscience, School of Medicine, University of Geneva, Geneva, Switzerland; Massachusetts Institute of Technology, United States of America

## Abstract

Dendritic spines are the main postsynaptic site of excitatory contacts between neurons in the central nervous system. On cortical neurons, spines undergo a continuous turnover regulated by development and sensory activity. However, the functional implications of this synaptic remodeling for network properties remain currently unknown. Using repetitive confocal imaging on hippocampal organotypic cultures, we find that learning-related patterns of activity that induce long-term potentiation act as a selection mechanism for the stabilization and localization of spines. Through a lasting N-methyl-D-aspartate receptor and protein synthesis–dependent increase in protrusion growth and turnover, induction of plasticity promotes a pruning and replacement of nonactivated spines by new ones together with a selective stabilization of activated synapses. Furthermore, most newly formed spines preferentially grow in close proximity to activated synapses and become functional within 24 h, leading to a clustering of functional synapses. Our results indicate that synaptic remodeling associated with induction of long-term potentiation favors the selection of inputs showing spatiotemporal interactions on a given neuron.

## Author Summary

In the central nervous system, excitatory contacts between neurons occur mainly on postsynaptic protrusions called dendritic spines. For decades, these structures have been considered static, and the adaptive properties of neuronal networks were thought to be only due to changes in the strength of neuronal connections. But recently, new imaging techniques used on living neurons revealed that spines and synapses are dynamic structures that undergo continuous turnover and can be formed or eliminated as a function of activity. The functional consequences of this structural remodeling, however, were still unknown. This work shows that application of learning related paradigms (such as induction of long-term potentiation or rhythmic activity) to hippocampal neurons allows them to operate a selection of synaptic inputs that show coincident activity. This is done through a competitive mechanism that promotes a selective stabilization of synapses activated by the learning paradigm and a replacement of non-activated inputs by new spines. Furthermore these new dendritic spines preferentially grow in close proximity to activated synapses and become functional. These findings provide evidence that learning related paradigms play a major role in shaping the structural organization of synaptic networks by promoting their specificity.

## Abstract 3

In hippocampal neurons, the induction of learning related patterns of activity leads to a selective stabilization of activated synapses. Moreover, new dendritic spines, which are functional, cluster in close proximity to activated synapses.

## Introduction

Integration of synaptic signals during learning processes is critical to the function of cortical networks. This processing is achieved through various mechanisms that involve generation of coincident rhythmic activity, induction of properties of plasticity such as long-term potentiation (LTP), but also growth of new protrusions and remodeling of synaptic networks [1–5]. The precise functional contribution of this structural remodeling to network properties remains unclear. In vitro experiments have demonstrated that LTP induction results during the next few hours in the growth of new filopodia and spines [6–9] which then rapidly become functional [10] and show all characteristics of morphologically mature synapses over the course of 24 h [11]. Also, work by several laboratories has shown that under in vivo conditions, spines and varicosities undergo a continuous turnover and replacement that vary in intensity as a function of development [12–16]. This process is further regulated by sensory activity, because under conditions of deprivation such as whisker trimming [17] or unbalanced activity such as chessboard whisker trimming [12,18], spine turnover increases, new spines form synapses and become stabilized, and others are eliminated. These experiments therefore clearly demonstrated that stable synaptic contacts can be removed or created de novo through experience, raising the possibility that synapse remodeling, together with Hebbian forms of plasticity, could contribute to information processing and learning [3,19]. It remains unclear, however, whether and how sensory activity regulates this synaptic remodeling and whether it could actually affect signal integration by the neuron and/or the network. Also, the rules and mechanisms determining which synapse should be removed or restructured and where new synapses should be created are unknown. These are important issues because both the number and localization of spines may greatly affect the properties of integration of synaptic responses by a neuron. Recent studies have shown that spatiotemporal clustering of synaptic currents on small or remote dendrites represents a critical aspect for the expression of plasticity and the contribution to neuronal firing [20–22]. Identification of the mechanisms that underlie spine and synapse remodeling is therefore critical to a better understanding of the processing properties of synaptic networks. We investigated these issues, using a repetitive imaging approach applied to hippocampal slice cultures, and analyzed how precisely learning-related activity patterns affected the long-term behavior of identified spines.

## Results

### LTP Induction Results in a Lasting Increase in Protrusion Turnover

**Figure 1:**
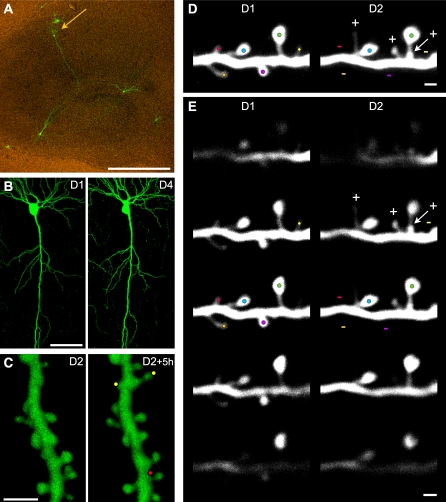
Illustration of the Experimental Approach (A) EGFP fluorescent CA1 pyramidal cell (arrow) in an 11 d in vitro organotypic hippocampal slice culture observed 3 d after transfection (scale bar: 100 μm). (B) Low magnification view of a CA1 pyramidal cell imaged on the first (D1) and fourth day (D4) of observation (scale bar: 50 μm). (C) 3D reconstructions of a dendritic segment imaged twice at 5-h intervals 24 h after LTP induction (see the axial rotation of the 3D reconstructed segments in Video S1). Note the appearance of two new protrusions (yellow dots) and disappearance of one (red dot) within this 5-h interval (scale bar: 2 μm). (D) Example of turnover analyses of z-stacks projections of raw images at observation day 1 (D1) and day 2 (D2). Color dots are placed on protrusions, one color per protrusion to facilitate their identification. At D2, three new protrusions are identified by a plus sign (+) and four lost protrusions by a minus sign (−) (scale bar: 1 μm). (E) Series of images from the z-stacks shown in (D) but at different z depths to illustrate the possibility to detect protrusions in the three dimensions. Color dots from (D) and + and − signs are reported on the z plan that is the most relevant for each protrusion (highest brightness). Note that the relative position of each protrusion along the *z*-axis is well conserved between observations (scale bar: 1 μm).

**Figure 2:**
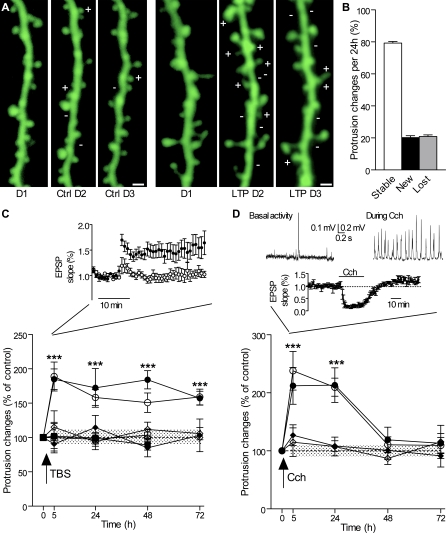
Lasting Increase in Protrusion Turnover Induced by Rhythmic Activity (A) Dendritic segments imaged on three consecutive days under control conditions (Ctrl, D1–D3) or before (D1) and following LTP induction (LTP, D1–D3; [+] new and [−] lost protrusions) (scale bars: 1 μm). (B) Proportion of stable, new, and lost protrusions (spines and filopodia) per 24 h under control conditions (*n* = 30 cells/1,627 protrusions). (C) Changes in protrusion turnover measured following LTP induction (TBS, circles; *n* = 17/916), or TBS + 100 μM D-AP5 (diamonds; *n* = 11/724), or low-frequency stimulation (0.3 Hz; squares; *n* = 6/183). Filled and empty symbols represent changes in new and lost protrusions, respectively. Data are plotted as percentage of control values with the shaded area representing the confidence interval. The insert shows the changes in EPSP slope measured in the CA1 area before and after LTP induction (filled circles; *n* = 13) and TBS + 100 μM D-AP5 (open circles; *n* = 7). (D) Same as in (C) but following theta activity (shown in the insert) induced by treatment with 10 μM Cch (circles, *n* = 11/723). Diamonds: 10 μM Cch + 100 μM D-AP5 (*n* = 8/639; ***p < 0.001, 2-way ANOVA with Bonferroni post-tests). The insert illustrates the spontaneous baseline activity of 1–3 Hz observed in one experiment and the increased 5–10 Hz activity induced by Cch. Below are shown the changes in EPSP slope measured during and after Cch treatment (*n* = 5).

Hippocampal slice cultures were transfected to express enhanced green fluorescent protein (EGFP) using a biolistic approach; we then monitored the behavior of identified protrusions (spines and filopodia) over several days following induction of learning-related activity patterns (Figure 1). For this, we used two different conditions that trigger LTP, a property believed to underlie learning mechanisms: first, we applied theta burst stimulation (TBS) to Schaffer collaterals, which triggers robust LTP, and second, we treated slice cultures for 20–60 min with carbachol (Cch, 10 μM), a cholinergic agonist, which, in the hippocampus and in slice cultures, triggers rhythmic activity in the theta and gamma range and induces a lasting synaptic enhancement (Figure 2C, inserts) [1,23]. In humans, these theta activities have been directly implicated in memory processes [24]. Fluorescent cells and dendritic segments were then imaged repetitively and the changes in protrusion number and long-term spine stability monitored (Figure 1A–1C) through analysis of single z-stack images (Figure 1D and 1E; see criteria in Materials and Methods). Control experiments with propidium iodide staining showed that transfection and repetitive confocal imaging of slice cultures did not alter cell viability over periods of weeks.

Analysis of protrusion turnover over periods of 3–8 d showed that the dynamics of synaptic networks is high at this developmental stage (11 d in vitro) with an average of 20.3% ± 1.1% new protrusions formed per 24 h and 20.8 ± 0.9% disappearing within the same period of time (Figure 2A and 2B). The other protrusions either remained stable without changes or underwent some sort of morphological transformations (16.2% ± 0.3% [25]). These values are in the range of those reported in vivo in the cortex of very young mice [12,14,15].

**Figure 3:**
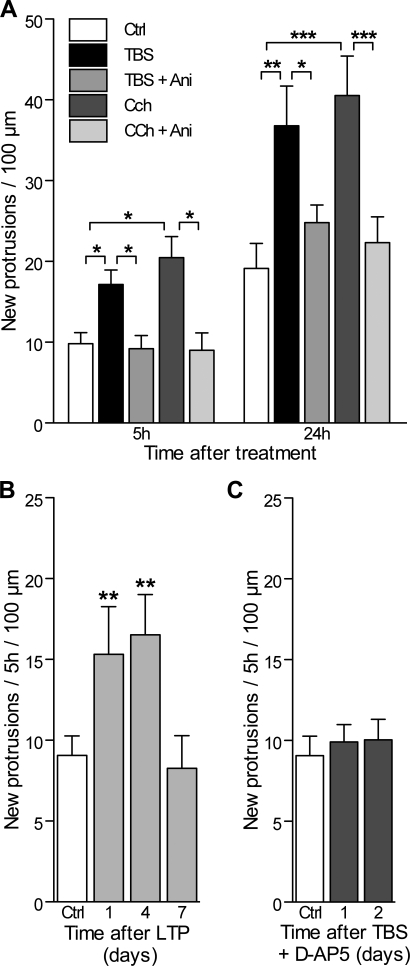
Increased Activity-Induced Protrusion Formation Is Protein Synthesis Dependent and D-AP5 Sensitive (A) Number of new protrusions (spines and filopodia) per 100 μm of dendritic length detected during the first 5 h and 24 h in control slice cultures (open columns), following TBS stimulation (black columns) or TBS applied in the presence of the protein synthesis inhibitor Ani (25 μM; grey columns; *n* = 6 cells/351 protrusions), as well as following Cch treatment (dark grey columns) and Cch together with Ani (light grey columns; *n* = 6 cells/307 protrusions). *p < 0.05, **p < 0.01; ***p < 0.001; two-way ANOVA with Bonferroni post-tests. (B) The number of new protrusions formed per 5 h and per 100 μm of dendritic length was measured under control conditions (*n* = 15 cells), and at 1, 4, and 7 d after LTP induction (*n* = 8). **p < 0.01, one-way ANOVA with Dunnett post-tests. (C) Rate of protrusion formation measured 1 and 2 d after TBS applied in the presence of 100 μM D-AP5 (*n* = 7).

Following theta burst activity we found that this basal turnover rate markedly increased. The effect was not short-lived [7,9], but the increase lasted for several days following a brief stimulation episode. This lasting increase in turnover rate was observed both following LTP induction by TBS (Figure 2C) and by Cch-induced rhythmic activity (10 μM; Figure 2D). The insert in Figure 2C shows the potentiation of the slope of evoked excitatory postsynaptic potentials (EPSPs) recorded in slice cultures following TBS. In Figure 2D, we illustrate the spontaneous baseline activity of 1–3 Hz observed under control conditions, the increased 5–10 Hz field activity recorded in the stratum pyramidale of CA3 during application of 10 μM Cch and the synaptic enhancement observed in the cornus ammonis 1 (CA1) region. The proportion of new and lost protrusions, which includes spines and filopodia, increased markedly under both conditions to values of 34% ± 6% and 43% ± 6% of new protrusions and 33% ± 2% and 44% ± 3% of lost protrusions over the first 24 h for TBS and Cch, respectively (see also Figure 3A). These changes reflected a similar increase in the formation of thin spines and filopodia, filopodia representing only a very small fraction of the new protrusions both under control conditions and after stimulation (4.3% ± 0.9%, *n* = 30 cells, control; 4.7% ± 1.4%, *n* = 17, LTP and 3.4% ± 1.6%, *n* = 17, Cch). Together, these experiments indicate a 70% and 115% increase in protrusion turnover rate following TBS or Cch treatment, respectively. To allow comparisons, the data obtained at the different observation times are expressed in Figure 2C and 2D as percentage of the basal rate of protrusion formation or loss observed under control condition. To test for the specificity of the effect, we then carried out the same experiments, but applied the N-methyl-D-aspartate (NMDA) receptor antagonist D(−)-2-amino-5-phosphonopentanoic acid (D-AP5; 100 μM) during the stimulation protocol or during the application of Cch. As shown in Figure 2C and 2D, D-AP5 specifically prevented the lasting increase in protrusion turnover under both conditions. As an additional control, we also analyzed hippocampal slice cultures stimulated in the same way at low frequency (0.3 Hz), but without induction of rhythmic activity. These controls showed no significant changes in turnover rate over time. Finally, we also tested whether this increase in protrusion turnover was dependent upon protein synthesis. For this, slice cultures were incubated in the presence of 25 μM anisomycin (Ani) and stimulated with either TBS or Cch. Under these conditions, both forms of potentiation were prevented (ratio of potentiation at 60 min: 1.13 ± 0.2, *n* = 6 and 1.08 ± 0.11, *n* = 3 for TBS and Cch, respectively) and, as shown in Figure 3A, no significant increase in the rate of protrusion formation or loss could be observed over the next 24 h. Note also that Ani treatment of cultures for 5 h without TBS or Cch stimulation did not affect the rate of formation and loss of protrusions over 24 h. These results thus indicated that the changes in protrusion turnover associated with induction of LTP lasted several days and included formation and elimination of spines and filopodia.

### Increased Protrusion Turnover Promotes the Replacement of Pre-Existing Spines by New Ones

**Figure 4:**
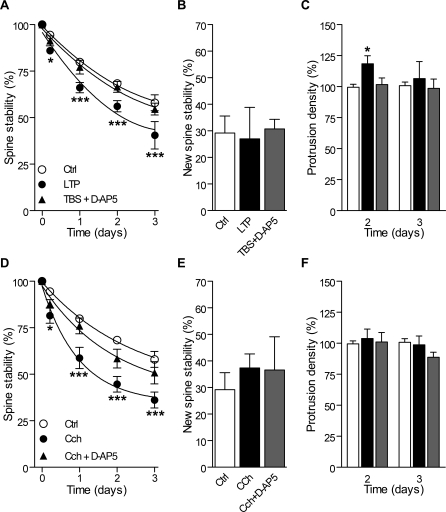
Changes in Spine Stability and Protrusion Density Induced by Theta Burst Activity (A) Stability of pre-existing spines measured as the proportion of initial spines still present 5 h, 1, 2, and 3 d later in control slices (*n* = 20/557), in slices following LTP induction (*n* = 8/220) and in slices stimulated with TBS + D-AP5 (*n* = 7/301). Filopodia were not considered because they are essentially transient. (B) Stability of the new spines formed during an interval of 5 h and still present 2 d later under control conditions, following LTP induction or TBS + D-AP5. (C) Changes in protrusion density (spines and filopodia) measured after 2 and 3 d in control slices (open columns), following LTP induction (black columns) and following TBS + D-AP5 (grey column). (D–F) Same as in (A), (B), and (C), respectively, but following Cch treatment (1 h, 10 μM; *n* = 11/328) or Cch + D-AP5 (*n* = 10/353). *p < 0.05, ***p < 0.001; two-way ANOVA with Bonferroni post-tests.

To assess these results further and test for possible changes in spine stability and/or occurrence of populations of transient spines or filopodia, we next analyzed protrusion growth each day over a period of 5 h, a period during which most new events can be detected [25]. Following LTP induction by TBS, the rate of protrusion formation expressed per 5 h and per 100 μm of dendritic segment increased by a factor of 2, and this for several days, an effect fully prevented by D-AP5 applied during the stimulation protocol (Figure 3B and 3C). We then also assessed spine stability, restricting the analysis to spines, since filopodia are essentially transient [25] and mostly disappeared within 24 h. The stability of pre-existing spines, calculated as the proportion of spines still present on consecutive days, significantly decreased following LTP induction (Figure 4A), a change also dependent upon NMDA receptor activation. The stability of the new spines formed within the first 5 h following LTP induction was however not affected (Figure 4B) and remained particularly low as under control conditions. Thus, LTP induction promoted protrusion growth, but also destabilization of pre-existing spines. Altogether, these different effects approximately cancelled each other, so that the protrusion density did not greatly vary; actually, a significant increase was only observed transiently 2 d following LTP induction (Figure 4C). A similar situation was observed following Cch treatment. Protrusion growth increased in association with a decrease in stability of pre-existing spines and no effect on the process of new spine stabilization or on protrusion density (Figure 4D–4F). With both types of experiments, therefore, the net effect on several days of this increased turnover was to promote the replacement of existing spines by new ones.

### Differential Stabilization of Activated and Nonactivated Spines by Rhythmic Activity

**Figure 5:**
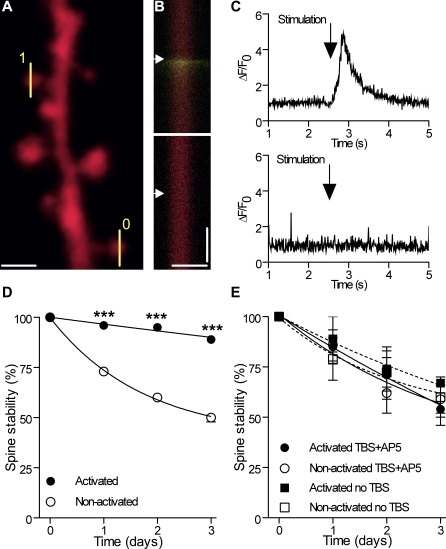
Differential Stabilization of Activated and Nonactivated Spines following Rhythmic Activity (A) Illustration of a dendritic segment with line scan analyses made on an activated (1) and a nonactivated (0) spine. (B) Line scans showing the mRFP and Fluo-4 channels obtained by analysis of the activated and nonactivated spines (scale bars: 1 μm; 1 s, arrows: stimulation of Schaffer collaterals). (C) Fluo-4 signals recorded in (B), expressed as ΔF/F_0_. (D) Differential long-term stability of activated (filled circles) and nonactivated spines (open circles) following LTP induction (*n* = 10 cells/62 activated and 130 nonactivated spines). (E) Same as in (D) but with TBS + 100 μM D-AP5 (*n* = 4 cells/36 activated [filled circles] and 44 nonactivated spines [open circles]). ***p < 0.001; two-way ANOVA with Bonferroni post-tests.

**Figure 6:**
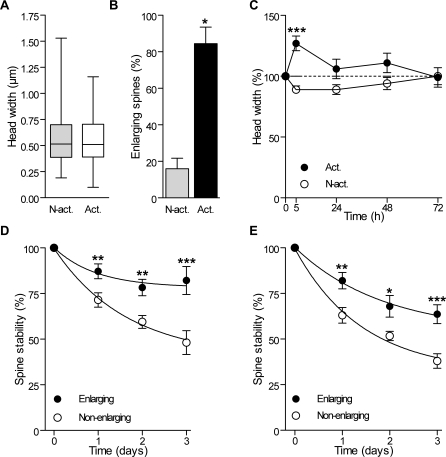
Differential Enlargement and Stability of Activated and Nonactivated Spines after Induction of Rhythmic Activity (A) Box plot distributions (min to max) of the head width of activated (Act; *n* = 98) and nonactivated spines (N-Act; *n* = 174) measured before TBS. (B) Proportion of activated and nonactivated spines that exhibited an enlargement 5 h after TBS (*n* = 4 cells/16 activated and 63 nonactivated spines). Spines were defined as enlarging if their head diameter increased by more than 0.1 μm in 5 h. (C) Changes in spine width of activated and nonactivated spines and expressed as percent of initial values (*n* = 4 cells/16 activated and 63 nonactivated spines). (D) Differential stability of spines that enlarged (filled circles) or did not enlarge (open circles) 5 h after LTP induction (*n* = 11 cells, 75 enlarging and 179 nonenlarging spines). (E) Differential stability of spines that enlarged (filled circles) or did not enlarge (open circles) 5 h after carbachol treatment (*n* = 4 cells/21 enlarging and 58 nonenlarging spines). *p < 0.05, **p < 0.01, ***p < 0.001; Mann-Whitney U-test (B), two-way ANOVA with Bonferroni post-tests (C, D, and E).

We then wondered how this increased spine remodeling could contribute to the specificity of the synaptic network and thus investigated whether it affected similarly activated and naive synapses. For this, we transfected pyramidal neurons with the red fluorescent dye monomeric red fluorescent protein (mRFP) [26], to visualize the structural changes in spine morphology, and costained them 3 d later with Fluo-4 AM, a calcium indicator, to identify spines activated by single pulse and TBS stimulation protocols (Figure 5A–5C, see also Material and Methods). Figure 5 illustrates the example of a dendritic segment with one spine that showed a clear increase in calcium fluorescence upon stimulation, while another one on the same segment remained silent. In all experiments carried out, we verified that spines activated by stimulation were always surrounded by other silent, nonactivated spines in order to exclude global activation effects. Also, we checked that analyses were done on spines of similar size (see Figure 6) and that the maximum calcium signal perfectly coincided with the center of the spine head. We then assessed the stability of activated and nonactivated spines for the next 3 d. Overall, with the stimulation pulses used under these conditions, on average, 36% of all spines tested on analyzed dendritic segments were found to be activated (*n* = 349 spines, 18 cells or segments). TBS was then applied to the same synapses using the same stimulation pulses in ten cells (62 activated and 130 nonactivated spines analyzed), which resulted in a differential effect on spine stability: activated spines showed a striking increase in stability in comparison to nonactivated spines present on the same dendritic portions (Figure 5D; *p* < 0.001). Nonactivated spines actually underwent pruning with regard to spines in nonstimulated slice cultures (Figure 5E; *p* < 0.05). Interestingly, this differential stabilization was prevented by D-AP5 applied during TBS (Figure 5E). We also verified that simple activation of spines without TBS did not affect the long-term stability of spines (Figure 5E, squares).

### Spine Activation Coincides with Spine Enlargement and Stabilization

Although for technical reasons we could not directly assess LTP in these stimulated spines, we found that most of them exhibited an enlargement of their head over the next 5 h. Several previous studies have indeed reported an enlargement of the spine head as a consequence of LTP induction [27–29] or used this criteria for identifying potentiated synapses [30]. In the group of 272 activated and nonactivated spines analyzed before TBS, there was no difference in mean head width (Figure 6A). However, when analyzed 5 h after TBS, most activated spines now exhibited an enlargement of their head, an effect not observed with nonactivated spines (Figure 6B). Interestingly, we also found that this differential enlargement was transient, as most activated spines reversed their size after 24 h and the differences with nonactivated spines then became nonsignificant (Figure 6C). Note, in addition, that the head width of nonactivated spines tended to become smaller after TBS and that the size of spine heads, when analyzed individually, showed regular fluctuations over consecutive days for both activated and nonactivated spines. A robust effect, however, was the close correlation observed between activated spines, spines that showed an enlargement 5 h after stimulation, and spines that became stabilized by activity. When using spine enlargement as a criteria to analyze spine stability, we found, as for activity, that enlarging spines exhibited the same differential stabilization (Figure 6D). Thus LTP induction is very likely to promote a long-term stabilization of potentiated synapses.

To verify whether Cch-induced rhythmic activity also produced the same selective stabilization process, we then analyzed how Cch treatment affected spine size. Analysis of 218 spines taken from nine dendritic segments showed that 34% of them exhibited enlargement of their head 5 h after Cch treatment. We then tested the stability of these spines over the next 2 d. As shown in Figure 6E, spines that enlarged as a result of Cch-induced rhythmic activity also became significantly more stable, while nonenlarging spines tended to be eliminated, showing the same differential behavior as after TBS-induced potentiation.

### Hot Spots for Spine Growth around Activated Synapses

**Figure 7:**
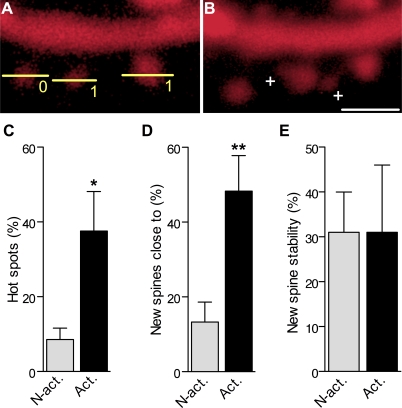
Clustering of Newly Formed Spines around Activated Synapses (A) Illustration of a dendritic segment with two spines (line scans, 1) that responded with a calcium signal to the stimulation and another spine (0) that did not. (B) Same segment imaged 24 h later showing the formation of two new protrusions (+) in close proximity (< 1.5 μm) of the activated spines (scale bar: 1.5 μm). (C) Proportion of activated and nonactivated spines that exhibited a new protrusion formed at < 1.5 μm (hot spot) within the next 48 h after TBS in seven experiments. (D) Proportion of new spines formed during the 48 h that followed TBS and appeared close to (< 1.5 μm) a nonactivated or an activated spine. (E) Stability of new protrusions measured as the proportion of protrusions formed within 5 h after TBS in close proximity to an activated or nonactivated spine and still present 48 h later. *p < 0.05; **p < 0.01, two-tailed unpaired *t*-test.

We then asked how these mechanisms could affect spine organization and distribution and analyzed whether newly formed spines could appear at specific hot spots. As shown in Figure 7A and 7B, we found that, indeed, newly formed spines tended to appear in close proximity to activated spines. In Figure 7C, we analyzed the proportion of activated versus nonactivated spines that had a new protrusion formed within a distance of 1.5 μm in the next 48 h (defined as hot spot). As indicated, almost half of activated spines had a new spine growing close by, something that did not occur with nonactivated ones. As shown by Figure 7D, we then examined all newly formed spines and asked how many actually grew close to an activated or a nonactivated spine. The results show that, again, about half of newly formed spines grew less than 1.5 μm from an activated spine, while only a small number of them grew close to a nonactivated spine, the others growing close to spines that could not be determined. The overall stability of newly formed spines was, however, not dependent on their localization (Figure 7E), because new spines generated close to or far from an activated spines showed the same probability of being present on subsequent days.

### New Spines Formed Consecutively to LTP Induction Are Functional

**Figure 8:**
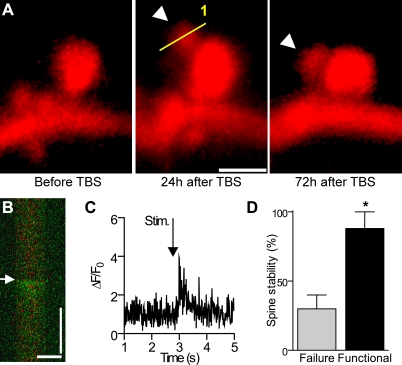
Functional Properties of Newly Formed Spines (A) Newly formed spine detected 24 h after application of TBS (line scan; scale bar: 1.5 μm) and still present 48 h later (arrowhead). (B) Line scan showing, superimposed, the mRFP and Fluo-4 channels obtained by analysis of the new spine illustrated in (A) and activated by electrical stimulation (arrow) 24 h after TBS (scale bars: 1 μm; 1 s). (C) Fluo-4 signal recorded in (B) following electrical stimulation (arrowhead) and expressed as ΔF/F_0_. (D) Stability of newly formed spines that responded by a calcium signal upon stimulation (functional, black column) or failed to respond (failure, grey column) and measured over 48 h later (*n* = 5 cells; *p < 0.05, Mann-Whitney U-test).

We then tested whether these newly formed spines became functional. For this, TBS was applied to an mRFP-transfected neuron, and the new spines formed within the next 24 h monitored by repetitive imaging and their functionality tested through loading with Fluo-4 AM and stimulation trials of Schaffer collaterals. Figure 8A shows an example of such a newly formed spine. Line scan analysis performed 24 h after TBS shows that this newly formed spine did indeed respond to stimulation through a calcium signal (Figure 8B and 8C), indicating that it was functional. Similar results were obtained in 30 spines out of 47 analyzed (*n* = 5 cells), indicating that a majority of them were functional. The mean ΔF/F_0_ ratio (i.e., [fluorescence − basal fluorescence]/basal fluorescence) at the peak of the calcium signal recorded in these experiments was 4.3 ± 0.8 (*n* = 30). For the other spines, it remains unclear whether they were silent or whether we simply could not activate them. We then asked whether the new functional synapses were also likely to be more stable than those that did not exhibit any calcium signal in response to stimulation. Of the 47 newly formed spines analyzed here, we found that the probability to persist for 48 h was 82% ± 12% for the 30 functional spines (*n* = 5), but only 30% ± 10% for the 17 nonactivated spines (Figure 8D), indicating that activity is a major criteria for long-term stability. Together these results indicate that LTP induction favored a clustering of new functional spines around activated spines, promoting in this way possibilities of spatiotemporal interactions between them.

##  Discussion

Together, these experiments provide evidence for an important new functional role of LTP-inducing activity in promoting a refinement of synaptic networks. Previous work in hippocampal slice cultures has shown that LTP induction is associated with two major types of structural remodeling. First, within minutes, potentiated synapses become larger and express larger and more complex postsynaptic densities [27–30], a change possibly associated with receptor expression and/or spine stabilization [31]. Second, within minutes to hours, LTP induction also results in the growth of new filopodia and spines [7,9,32], which then eventually become functional synapses [8,10,11,25]. These in vitro data are consistent with other in vivo experiments indicating that sensory deprivation or unbalanced activity does indeed affect cortical spine turnover and promote formation of new synapses [17,18,33]. Here we add three new pieces of information providing a novel, important function for structural plasticity: namely, to operate as a selection process for the long-term stability of synaptic contacts and the promotion of spatiotemporal interactions between spines.

First, we provide the first (to our knowledge) direct evidence that spines stimulated with LTP-inducing protocols are selectively stabilized over periods of several days. Although LTP could not be directly assessed together with repetitive imaging, we find that stabilization occurred specifically at spines stimulated with TBS and not at nonstimulated spines. Also, stabilized spines did exhibit an enlargement of the head at 5 h, a characteristic now demonstrated to be directly associated to LTP by several recent studies [27–30]. Finally, spine enlargement and spine stabilization were both D-AP5 sensitive and protein synthesis dependent. It seems therefore likely that the stabilization of stimulated synapses revealed here represents a central mechanism for the persistence of potentiated synapses.

The second new feature uncovered by these experiments is that LTP is not only associated with a short-term increase in protrusion growth, but a lasting, enhanced turnover that affects pre-existing spine stability, probably through competition mechanisms. Consistent with previous data [7,9], protrusion growth initially tended to predominate over spine loss, leading to a transient increase in spine or protrusion density. However, all together, LTP mainly affected turnover, resulting not only in protrusion growth, but also in an increased loss and destabilization of spines, which, importantly, specifically affected nonstimulated spines. The net effect of LTP over several days was therefore to promote the replacement of nonactivated spines by new ones. This selective destabilization of nonactivated spines was quantitatively significant, because in these experiments more than 10% of the spines of the neurons were actually replaced. Accordingly, regular occurrence of activity susceptible to induce LTP works as a selection mechanism leading to a progressive stabilization of inputs showing coincident activity, increasing in this way the coherence of the synaptic information provided to the neuron and reducing background noise.

The last important finding of these experiments is that newly formed protrusions do not appear just anywhere, but tend to cluster around activated spines. These new spines also become functional, and when functional, tend to remain stable. Together with the evidence that LTP induction is facilitated between spines located close to each other [30], this result indicates that LTP will actually promote the creation of hot spots of functional synapses. This provides therefore a means to promote spatiotemporal clustering of synaptic signals, a property recently shown to be critical for determining the characteristics of plasticity and processing at synapses on small or remote dendrites [20–22].

At the molecular level, an interesting implication of these results is that LTP mechanisms are likely to involve specific changes that could directly affect spine stability. Spine enlargement has been previously proposed to reflect this process [31] and, consistent with this idea, we indeed found that activated spines did enlarge 5 h after stimulation. Curiously, however, this effect did not seem to remain stable over 24 h, and analyses of spine head width suggest that most spines regularly exhibit significant variations of their size [30]. It could be, therefore, that stability is not only reflected in the size of the spine, but is linked to the expression of specific molecules. The current evidence indicating a contribution of protein synthesis to the long-term changes in synaptic strength and to the regulation of spine turnover as reported here could actually suggest such a mechanism [34]. In order to become stable, activated spines would need to accumulate the machinery required for protein synthesis [35] and/or express specific molecules conferring stability to the synaptic contact.

Taken together, the mechanisms reported here provide a new framework for understanding how the specificity of cortical networks may progressively develop. These results might be particularly important during critical periods when refinement of connections represents a major process shaped by rhythmic activity and dynamic regulations between excitatory and inhibitory transmission [36]. This network plasticity might, however, also contribute in the adult and provide the functional rules underlying the spine dynamics described in association with sensory activity [18] or following brain damage [37]. Together the synaptic mechanisms described here certainly point to the important role played by structural plasticity in association to Hebbian changes in synaptic strength for the refinement and specificity of cortical networks.

## Materials and Methods

### Slice cultures and transfection.

Transverse hippocampal organotypic slice cultures (400 μm thick) from 6- to 7-d-old rats were prepared as described [38] using a protocol approved by the Geneva Veterinarian Office (authorization 31.1.1007/3129/0) and maintained for 11–18 d in a CO_2_ incubator at 33 °C. Transfection was done either with a pc-DNA3.1-EGFP or a pCX-mRFP1 [39] plasmid using a biolistic method (Helios Gene Gun, Bio-Rad) 2–3 d before the first observation. Fluorescence usually started to be expressed after 24–48 h and then remained stable for at least 15 d.

### Electrophysiology.

For electrophysiological recordings, slice cultures were maintained at 32 °C in an interface chamber under continuous perfusion as described [40]. EPSPs were evoked by stimulation of a group of Schaffer collaterals and recorded in the stratum radiatum of the CA1 region with pipettes filled with medium. Potentiation was analyzed by measuring EPSP slopes expressed as percent of baseline values using an acquisition program written with Labview. LTP was induced by TBS (five trains at 5 Hz composed each of four pulses at 100 Hz, repeated twice at 10-s intervals). As controls, we used slice cultures stimulated at low frequency (0.3 Hz) and recorded in the same manner as well as slice cultures stimulated with TBS but in the presence of 100 μM D-AP5. In these experiments, D-AP5 was only applied for 30 min during application of TBS. Cch treatment was applied for 20–60 min at a concentration of 10 μM with or without concomitant application of D-AP5. The protein synthesis inhibitor was Ani applied 1 h before TBS or Cch treatment at a concentration of 25 μM and then maintained for 3 h.

### Confocal imaging.

Short imaging sessions (10–15 min) of transfected slices were carried out with an Olympus Fluoview 300 system coupled to a single (Olympus) and a two-photon laser (Chameleon; Coherent) as described [25]. Laser intensity in all these experiments was kept at the minimum and acquisition conditions maintained mostly unchanged over the different days of observation. Control experiments showed that transfection and repetitive confocal imaging of slice cultures did not alter cell viability over periods of weeks.

We focused on dendritic segments of about 35 μm in length and located between 100 and 300 μm from the soma on secondary or tertiary dendrites using a 40× objective and a 10× additional zoom (final resolution: 25 pixels per micron; steps between scans: 0.4 μm; Figure 1). We did not find differences in protrusion turnover within the limits of these dendritic locations. For calcium imaging of spine activity, transfected cells were additionally loaded with the cell-permeable calcium indicator Fluo-4 AM (F-14201, Invitrogen). For this, 50 μg of Fluo-4 AM was dissolved in 10 μl Pluronic (F-127, Invitrogen) and then diluted in 90 μl of standard pipette solution (150 mM NaCl, 2.5 mM KCl, 10 mM Hepes) for a final dye concentration of 500 μM. A standard patch pipette was then filled with 10 μl of dye solution and placed at a distance of about 10 μm from the soma of a mRFP1-expressing CA1 pyramidal cell. Dye was ejected by short pulses of pressured air at a frequency of three per minute during one-half hour. Calcium transients in 10–26 identified spines per dendritic segment were then recorded using line scans through the spine heads obtained during application of stimulation pulses to Schaffer collaterals. These pulses were of identical intensity and duration to those used for subsequent induction of LTP. Confocal aperture was set to the minimum during line scans, and matching with the mRFP fluorescence in the red channel was systematically checked. For each spine tested, calcium transients evoked by two or three consecutive stimulation pulses were recorded, and spines were determined as activated whenever the fluorescence signal increased by more than 20% over background in any of the recordings. In average, 36% of all spines tested corresponded to these criteria with the stimulation pulses used. To avoid biases, we then also verified that the size distribution of the spine heads did not differ between spines classified as activated and nonactivated (0.56 ± 0.02 μm versus 0.58 ± 0.02 μm, respectively; Figure 6A).

### Image analysis.

In this study we refer to protrusions, whenever analyses were carried out by considering filopodia and spines. Filopodia were defined as protrusions devoid of enlargement at the tip, while we classified as spines all protrusions exhibiting an enlargement at the tip. All turnover and stability analyses were carried out on single z-stacks of raw images (Figures 1E and S1) using a plug-in specifically developed for OsiriX software (http://www.osirix-viewer.com). The measures of turnover were carried out by analyzing all protrusions, i.e., filopodia and spines. We counted as new protrusions all new structures (spines or filopodia) appearing between two observations (5 or 24 h) and characterized by a length of >0.4 μm. All filopodia were counted as separate protrusions. We also counted spines located behind each other on z-stacks whenever distinction was possible (Figures 1E and S1). For disappearances, we counted all protrusions (spines and filopodia) that could no longer be identified on the next observation. Dubious situations due to possible changes in protrusion shape, size, or orientiation were discarded, but overall accounted for only a small number of cases (less than 1%). To further ensure reliability of analyses, all measurements of spine turnover and stability were carried out blind by two experimenters. Comparisons of the analyses made in this way showed variations in the results that were less than 3%. Furthermore, we used high numbers of *n* for both cells and spines, and labeled all new or lost protrusions directly on the raw data (Figure 1E) to allow multiple checks.

Due to the lack of survival of filopodia on several days, stability analyses carried on 48 or 72 h periods only included pre-existing spines, i.e., spines present at the beginning of the experiment. For analyses of spine width, we measured the maximum diameter of the spine head on individual z-images, setting the fluorescence level on the levels obtained in the dendrite. Situations that did not allow a precise spine head width measurement (two spine heads overlapping each other on the same z sections) were excluded. Calcium fluorescence intensities were acquired and analyzed with Fluoview software (FV300, Olympus). Note that for illustration purposes, images presented in the figures are maximum intensity projections of z stacks, further treated with a Gaussian blur filter. All statistics are given with the standard error of the mean. Normality was tested for each distribution (D'Agostino and Pearson test), and α was set to 5% for all tests.

## Supporting Information

Figure S1 Z-Stack Details from the Dendrite Illustrated in Figure 2A (A) Z-stacks projections of raw images obtained before (D1), 24 h (LTP-D2) and 48 h (LTP-D3) after LTP induction. Color dots or triangles are placed on protrusions, one color per spine, to facilitate protrusion identification. On day 2 (LTP-D2), three new protrusions are identified by a plus sign (+) and a color triangle; three lost protrusions by a minus sign (−). On day 3 (LTP-D3), two new protrusions are identified by a plus sign (+) and one lost protrusion by a minus sign (−).
(B) Series of images from the same stacks as in (A) but at different z steps to illustrate that scrolling through the *z*-axis allows a correct discrimination between protrusions. Color dots, triangles, and plus and minus signs from (A) are reported on the z level that is the most relevant for each protrusion (highest brightness) (scale bars: 1 μm).
(6.57 MB TIF)

Video S1 3D Rotating Movie of the Dendritic Segment Illustrated in Figure 1C (2.38 MB MOV)

## Abbreviations

ΔF/F_0_: (fluorescence − basal fluorescence)/basal fluorescence
Ani: anisomycin
CA1: cornus ammonis 1
Cch: carbachol
D-AP5: D(−)-2-amino-5-phosphonopentanoic acid
EGFP: enhanced green flourescent protein
EPSP: excitatory postsynaptic potential
LTP: long-term potentiation
mRFP: monomeric red fluorescent protein
NMDA: N-methyl-D-aspartate
TBS: theta burst stimulation
